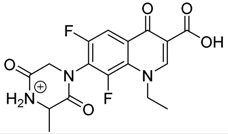# Development and Validation of Stability-Indicating HPLC Methods for the Estimation of Lomefloxacin and Balofloxacin Oxidation Process under ACVA, H_2_O_2_, or KMnO_4_ Treatment. Kinetic Evaluation and Identification of Degradation Products by Mass Spectrometry

**DOI:** 10.3390/molecules25225251

**Published:** 2020-11-11

**Authors:** Barbara Żuromska-Witek, Paweł Żmudzki, Marek Szlósarczyk, Anna Maślanka, Urszula Hubicka

**Affiliations:** 1 Department of Inorganic and Analytical Chemistry, Faculty of Pharmacy, Jagiellonian University Medical College, Medyczna 9, 30-688 Kraków, Poland; barbara.zuromska@uj.edu.pl (B.Ż.-W.); m.szlosarczyk@uj.edu.pl (M.S.); anna.maslanka@uj.edu.pl (A.M.); 2 Department of Medicinal Chemistry, Faculty of Pharmacy, Jagiellonian University Medical College, Medyczna 9, 30-688 Kraków, Poland; pawel.zmudzki@uj.edu.pl

**Keywords:** balofloxacin, lomefloxacin, oxidation studies, kinetic evaluation, HPLC

## Abstract

The oxidation of lomefloxacin (LOM) and balofloxacin (BAL) under the influence of azo initiator of radical reactions of 4,4′-azobis(4-cyanopentanoic acid) (ACVA) and H_2_O_2_ was examined. Oxidation using H_2_O_2_ was performed at room temperature while using ACVA at temperatures: 40, 50, 60 °C. Additionally, the oxidation process of BAL under the influence of KMnO_4_ in an acidic medium was investigated. New stability-indicating HPLC methods were developed in order to evaluate the oxidation process. Chromatographic analysis was carried out using the Kinetex 5u XB—C18 100A column, Phenomenex (Torrance, CA, USA) (250 × 4.6 mm, 5 μm particle size, core shell type). The chromatographic separation was achieved while using isocratic elution and a mobile phase with the composition of 0.05 M phosphate buffer (pH = 3.20 adjusted with *o*-phosphoric acid) and acetonitrile (87:13 *v/v* for LOM; 80:20 *v/v* for BAL). The column was maintained at 30 °C. The methods were validated according to the ICH guidelines, and it was found that they met the acceptance criteria. An oxidation process followed kinetics of the second order reaction. The most probable structures of LOM and BAL degradation products formed were assigned by the UHPLC/MS/MS method.

## 1. Introduction

The stability of drugs is the one of the basic requirements, which is closely related to the efficiency of pharmacological action and safety of therapy. Changes in the structure of a chemical compound and physicochemical properties may lead to a decrease in the therapeutic value of the drug through disturbances in the absorption process or a decrease in pharmacological activity or even increase in its toxicity. Therefore, the chemical stability of pharmaceutical substances is a significant problem that affects the safety and efficiency of the pharmaceutical preparation. The stability of the pharmaceutical active substance can be tested by stress tests, long-term, intermediate, and accelerated stability studies [1]. Oxidation reactions are one of the two most common mechanisms of drug degradation in pharmaceutical systems [2,3,4]. The reactions are typically autoxidative and its mechanism consists of the formation of free radicals. Radical-initiated chain reactions start with an initiation phase (formation of radicals is rate-limiting), followed by a propagation phase and termination phase [1,5]. The initiators of the oxidation chain reactions of the active substance may be chemical compounds of various origin. There are many examples, such as: peroxy (ROO^•^) and oxy (RO^•^) radicals derived from the thermal haemolytic decomposition of organic peroxides or photochemical reactions; heavy metal impurities from the manufacturing process; radiolysis or ozonolysis products. Described oxidation agents are often contaminants of Active Pharmaceutical Ingredients (APIs), excipients, or environmental pollutants [3,4]. ICH Q1A (R2) guidelines described conditions of stress tests which depends on the drug substance and the type of medicinal product [6]. However, the information is general, there are no recommended oxidants or conditions for carrying out the oxidation process, and therefore there is a freedom in the planning of stress tests that a scientist can afford for scientific purposes [1]. One of the agents mentioned above is hydrogen peroxide, which is widely used to study the oxidation of medicinal substances in stress testing. As a result of the oxidation of the API with the use of hydrogen peroxide, degradation products are often formed, which can be formed during long-term decomposition as minor impurities. This is not surprising that hydrogen peroxide and organic hydroperoxides belong to autooxidation products. Other oxidizing agents used in stress testing are metal ions, oxygen, and azo radical initiators, e.g., 2,2′-azobisisobutyronitrile (AIBN) [4,7,8]. In the case of hydrogen peroxide, it is recommended to conduct the reaction at room temperature, because raising the temperature is associated with the risk of the formation of undesired hydroxyl radicals 2OH**^•^**. Hydrogen peroxide is often used, but it does not allow for the prediction of reactions involving molecular oxygen, which is a paramagnetic diradical in its basic state; therefore, radical reactions are often used as a good model for drug oxidation process. In addition, stress testing using H_2_O_2_ often leads to secondary reactions of degradation of primary degradation products, thus hindering the interpretation of the obtained results. Stress tests using hydrogen peroxide are useful for examining medicinal products in which hydrogen peroxide occurs as an expected contamination of excipients [2,4,9,10,11,12,13]. Therefore, the test in the presence of H_2_O_2_ should always be carried out in parallel with the radical initiator test, as it provides complementary information.

The initiators of radical reactions are azo compounds, including 2,2′-azobisisobutyronitrile (AIBN) and 4,4′-azobis(4-cyanopentanoic acid) (ACVA), which are widely used in forced stress procedures [4,14,15]. Azo initiators have many advantages over standard organic peroxides, such as the fact that they form no oxygenated byproducts, only generating C-centered radicals, which are low energy and very selective [16]. Although AIBN is used in organic synthesis usually at temperatures between 60 °C and 80 °C, its oxidation is carried out at a much lower temperature of 40 °C, as oxidation conditions should simulate long-term degradation and allow for the isolation of labile degradation products, such as hydroperoxides that are destroyed at higher temperatures [4]. Boccardi et al. proposed azonitrile-based radical initiators to study the autooxidation mechanism of pharmaceuticals, demonstrating that azonitrile-based compounds could produce primary oxidation/degradation products that were observed by long-term storage of a drug and pharmaceutical forms [4,17]. Waterman et al. [18] and Hovorka et al. [8] in the review of the oxidative degradation of pharmaceuticals have assumed that azonitrile-based radical initiators were useful to predict oxidation products by autooxidation. Although many factors, such as: heat, light, and metal catalysis promote initiation, initial radical formation by these methods is difficult to control experimental parameters of the forced stress procedure, especially in complex pharmaceutical formulations. For mechanistic purposes, the utilization of azo initiators allows for the quantitation of initial radical yields and it affords reproducible results with slightly elevated temperature or under controlled photolytic conditions. Alsante et al. recommended that free radical-based oxidation experiments using azonitrile-based radical initiators should be conducted at all stages of development and provided the information how to carry out forced degradation studies while using AIBN and ACVA [19]. Nelson et al. described the azonitrile initiator solutions as a good predictive model of the major oxidative degradation products that were observed in pharmaceutical formulations. At low to moderate initiator concentrations, the degradation product distributions and degree of reactivity were similar for samples that were stored in ambient and pressurized oxygen environments [14].

One of the specific, most promising, and intensely studied drug groups in modern antibiotic therapy, which well-known from the susceptibility for the degradation process are fluoroquinolones (FQs) [20,21,22]. This group occupy a very important place in modern medicine due to the broad spectrum of antibacterial activity, the possibility of oral and parenteral administration, good absorption, achieving high therapeutic concentrations in tissues and body fluids, rapid elimination from the body, chemical, and biological stability [23]. Lomefloxacin (LOM) and balofloxacin (BAL) belong to the second and third generation of FQs class antibiotic, respectively. Balofloxacin and lomefloxacin inhibit both the synthesis of bacterial DNA by the suppression of the enzyme DNA gyrase. In addition, the bactericidal action of LOM and BAL results from the inhibition of topoisomerase IV, which is needed for the transcription and replication of bacterial DNA [24]. The degradation studies on LOM have been performed while using different oxidation agents: H_2_O_2_/UV, alkaline permanganate, permanganate in acidic medium and catalytically activated persulfate, in few cases the degradation products were separated and identified by LC-MS [25,26,27,28]. In the available literature, there is no information regarding hydroxyl-radical induced oxidation of BAL or in the presence of H_2_O_2_, although several studies are focused on its photooxidation process [29,30].

The aim of this study was to evaluate the oxidation of LOM and BAL under the influence of azo initiator of radical reactions of ACVA and hydrogen peroxide, and to compare both of the processes. The oxidation of BAL under the influence of KMnO_4_ in an acidic medium has not been extensively described in the literature, so it was decided to also investigate this process. In order to achieve the above goals, new HPLC-DAD methods allowing for the determination of LOM and BAL next to oxidation products were developed and validated. The kinetic evaluation of the oxidation process was performed, and the degradation products were identified by the UHPLC-MS/MS method. It seems that the research we have undertaken has a cognitive and application value, because, in the available literature, we have not found comprehensive research on the oxidative degradation of LOM and BAL in the presence of oxidants that were selected by us.

## 2. Results and Discussion

### 2.1. Optimization of Chromatographic Conditions

The usefulness of mobile phases containing 0.05 M phosphate buffer with pH = 3.20 and acetonitrile in various proportions (80:20, 85:15, 87:13, *v/v*) was checked in order to develop an appropriate chromatographic method to separate BAL or LOM from their oxidation products.

The solutions of the tested FQs after 48 h of incubation at 50 °C in 10 mM ACVA, after 72 h incubation in 3% H_2_O_2_ and after 1 h of incubation in acidic medium with the presence of KMnO_4_ were used for the analysis. On the basis of the obtained chromatographic parameters, such as peak symmetry, resolution factor, and retention time, the buffer/acetonitrile mixtures 80:20 (*v/v*) and 87:13 (*v/v*) were selected in order to evaluate the oxidation of BAL and LOM, respectively.

### 2.2. Method Validation

**Figure 1:**
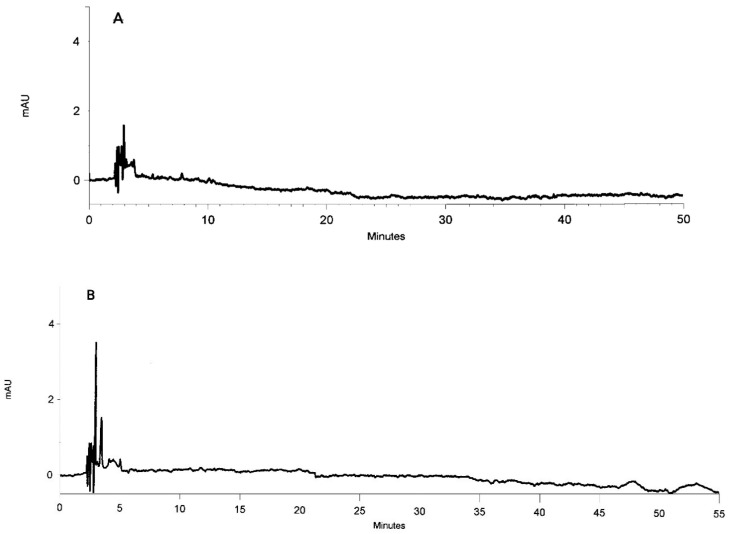
Chromatograms of the 4,4′-azobis(4-cyanopentanoic acid) (ACVA) control samples after incubation for 48 h at 60 °C recorded at: (**A**)—295 nm, (**B**)—280 nm.

The developed HPLC methods meet the acceptance criteria for the specificity. In the proposed chromatographic conditions, well shaped peaks were obtained for examined FQs and oxidation products. The satisfactory resolution was also guaranteed both for peaks of main substances and degradation products. The values of the separation factors obtained for the closest adjacent degradation product and the tested FQ were greater than 2.5. Moreover, at the analytical wavelength, the ACVA control sample did not give additional peaks in the chromatogram, which could interfere with peaks of oxidation products (Figure 1).

As a result of the regression analysis, it was stated that the obtained parameters fitted well to the linear model of relationship in studied concentrations range. The correlation coefficients and obtained determination coefficients were greater than 0.99. The y-intercepts of the linear equation for BAL and LOM were statistically insignificant. *p*-values of the Shapiro-Wilk normality test were greater than 0.05 what confirms the normal distribution of the residuals. Linearity range was obtained in the wide concentration range (0.05–0.18 mg·mL^−1^ for LOM and 0.05–0.26 mg·mL^−1^ for BAL). Table 1 presents all of the described regression analysis results.

Based on the LOD and LOQ values, which were found to be from 0.0138 to 0.0419 mg·mL^−1^ and from 0.0081 to 0.0172 mg·mL^−1^, for LOM and BAL, respectively, it was assumed that the method was characterized by a good sensitivity. Good precision and intermediate precision with %RSD less than 0.83% were obtained (Table 1). A slight change (± 5% from the optimal value) in the composition of the mobile phase caused significant changes in the quality of the chromatographic separation that were related to the appearance and shape of the obtained peaks. Therefore, it is important to maintain a constant ratio of buffer and acetonitrile in a mobile phase.

### 2.3. Oxidation of Examined FQs

The data on the influence of the solvent on the oxidation process with ACVA have been found in the available literature [31]. Taking the above information into account, a methanolic and aqueous solutions of LOM were prepared and analyzed. Due to the fact that BAL is insoluble in water, only the methanol solution was prepared for testing.

The oxidation of BAL and LOM using the radical initiator ACVA has been tested in solutions incubated at 40 °C, 50 °C, and 60 °C. Likewise, the oxidation process of BAL and LOM after reaction with commonly used hydrogen peroxide at room temperature (RT) was examined.

The obtained results demonstrated that the course of the oxidation process depends on the type of FQs, incubation time, temperature, and the oxidizing agent used. Increasing the incubation time and temperature resulted in an increase in the degradation of the tested compounds.

**Figure 2:**
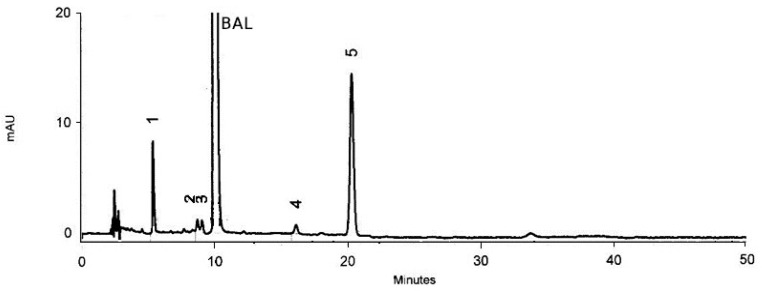
Chromatogram of balofloxacin (BAL) after oxidation by ACVA at 40 °C after incubation for 144 h.

**Figure 3:**
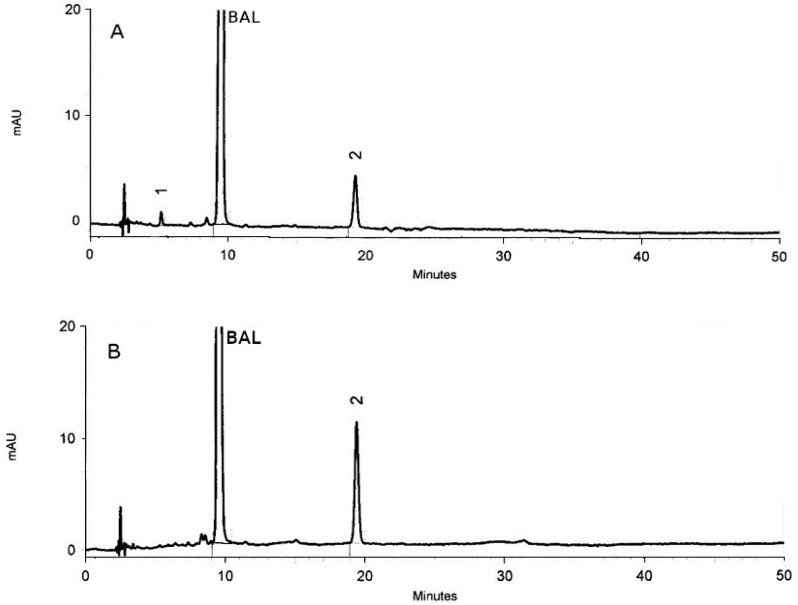
Chromatograms of BAL after oxidation by ACVA at 50 °C. Incubation time: (**A**)—36 h, (**B**) —48 h.

**Figure 4:**
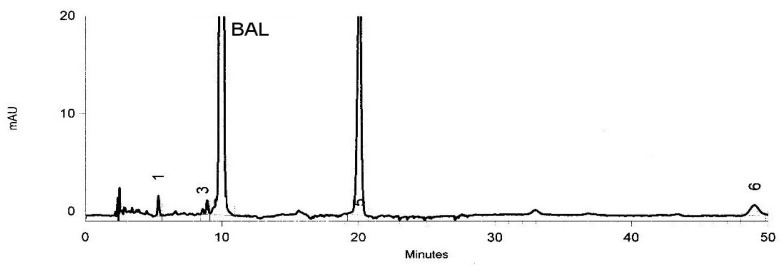
Chromatogram of BAL after oxidation by ACVA at 60 °C. Incubation time—48 h.

The percentage of BAL degradation under the influence of ACVA after 144 h of incubation at 40 °C was 16.31%, after 48 h of incubation at 50 °C was 18.34% and after 48 h of incubation at 60 °C was 36.76%. The oxidation of BAL by ACVA led to the formation of two main degradation products (t_R_ ≈ 5.40 min and 20.30 min) and the four others with a small amount (<0.7%) (Figure 2, Figure 3 and Figure 4).

The percentage of LOM degradation (methanolic solution) at 40 °C after 216 h of incubation was 20.62%, at 50 °C after 96 h—36.76%, while at 60 °C a similar percentage of degradation (38.55%) was already observed after 48 h of incubation.

**Figure 5:**
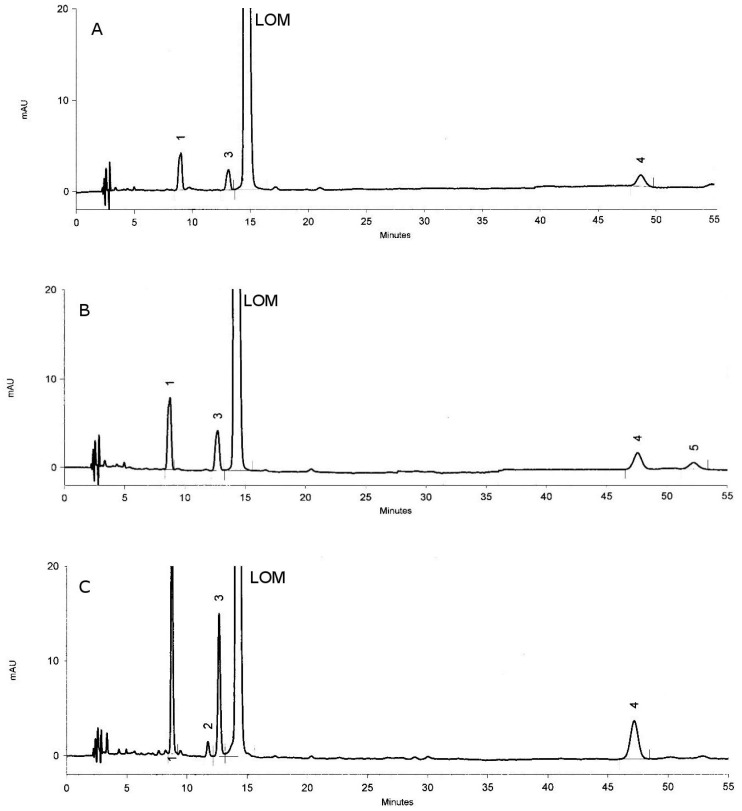
Chromatograms of methanolic solution of lomefloxacin (LOM) after oxidation by ACVA: (**A**)—40 °C and 216 h of incubation, (**B**)—50 °C and 96 h of incubation, (**C**)—60 °C and 48 h of incubation.

The use of ACVA and presence of methanol resulted in the formation of three main LOM products (t_R_ ≈ 8.78 min, 12.70 min, and 47.30 min). Moreover, two additional oxidation products (amount < 0.8%) with retention times of 11.74 min and 52.26 min were registered at temperature 60 °C and 50 °C, respectively. Figure 5 shows exemplary chromatograms.

**Figure 6:**
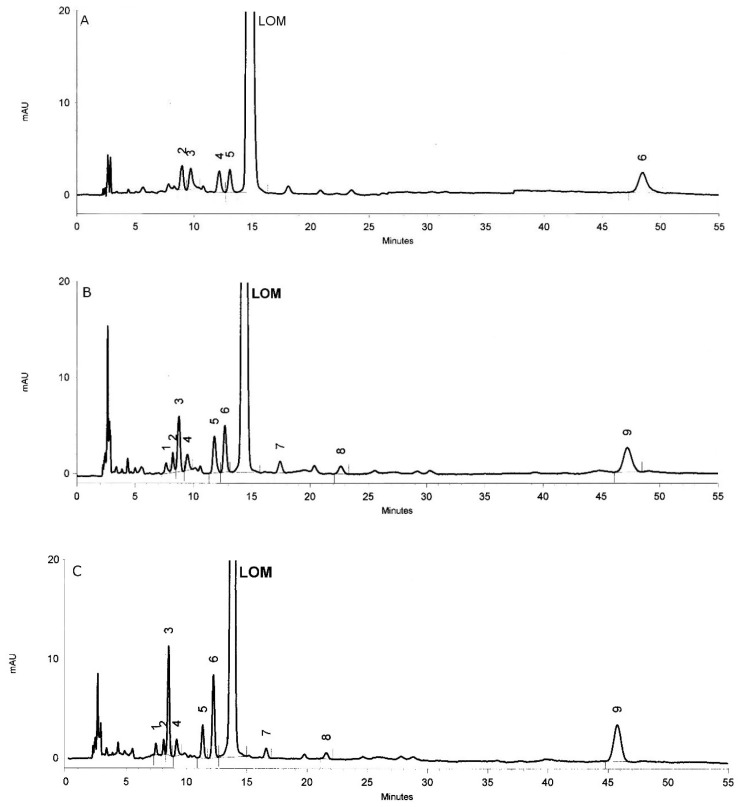
Chromatograms of water solution of LOM after oxidation by ACVA: (**A**)—40 °C and 96 h of incubation, (**B**)—50 °C and 72 h of incubation, (**C**)—60 °C and 36 h of incubation.

In turn, the results of oxidation of LOM (aqueous solution) by ACVA showed that the absence of methanol in the solution accelerates the process and causes the formation of as many as eight degradation products. After 96 h of incubation at 40 °C, the percentage of degradation of LOM was 14.28%, after 72 h at 50 °C, 43.96%, and at 60 °C, after 36 h, 34.17% (Figure 6).

**Figure 7:**
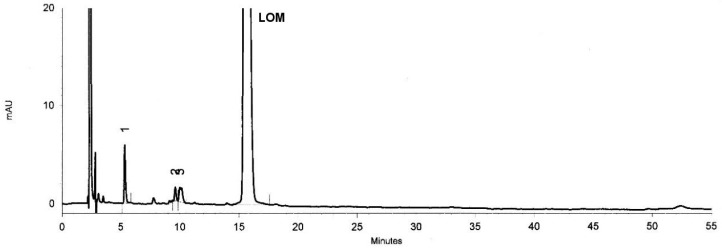
Chromatogram of LOM after oxidation by H_2_O_2_ after incubation for 168 h.

**Figure 8:**
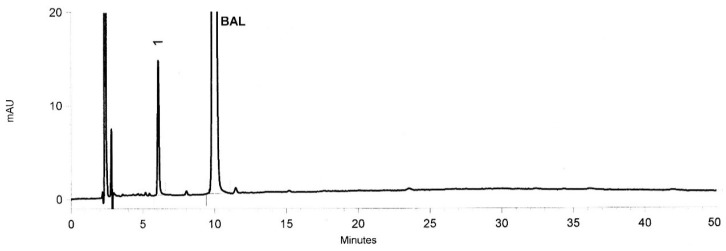
Chromatogram of BAL after oxidation by H_2_O_2_ after incubation for 72 h.

Studies with 3% H_2_O_2_ showed that the degradation of LOM was slower than BAL, after 96 h of incubation its degradation was 5.88% and after 168 h incubation it increased to 8.13%. In contrast, the oxidation degradation of BAL after 96 h was 17.3%. The oxidation process of LOM led to the formation of one of the main degradation products (t_R_ = 5.34 min) and the two others with a small amount (t_R_ = 9.61 min and 10.0 min) (Figure 7). Only one product was formed as a result of the oxidation of BAL (Figure 8).

In our previous work, we described the oxidation of LOM under the influence of KMnO_4_ [27], but we did not conduct such studies for BAL. Therefore, the oxidation process of BAL under the influence of KMnO_4_ in an acidic environment at RT was additionally investigated.

**Figure 9:**
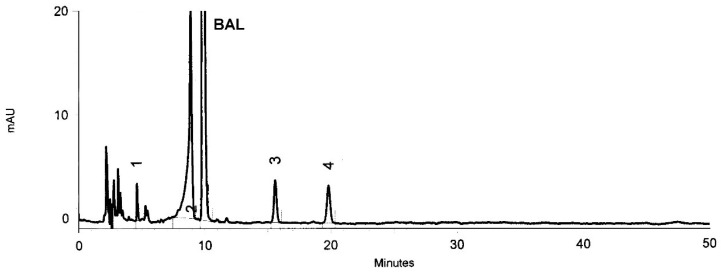
Chromatogram of BAL after oxidation by KMnO_4_ in the acidic medium after incubation for 1 h at RT.

The oxidation process of BAL under the influence of KMnO_4_ was very fast and, after one hour of incubation, the percentage of FQ degradation was 56.26. The process led to the formation of one major degradation product (t_R_ = 9.12 min) and three smaller ones (t_R_ = 4.66; 15.60; 19.81 min) (Figure 9).

### 2.4. Kinetic Evaluation

**Figure 10:**
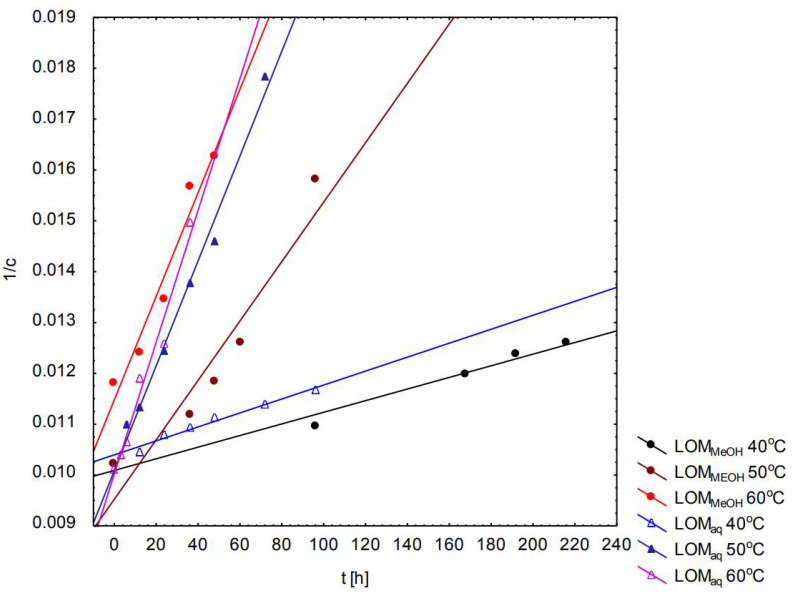
The 1/c = f(t) graph of oxidation of aqueous and methanolic LOM solutions under the influence of ACVA.

**Figure 11:**
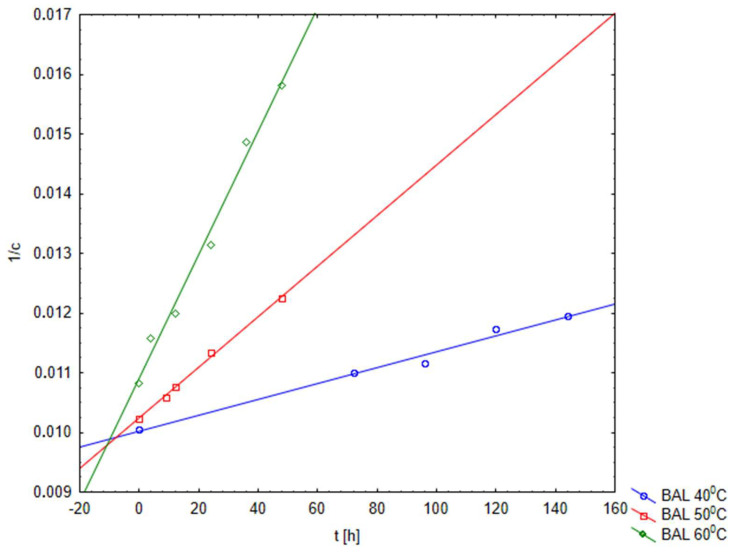
The 1/c = f(t) graph of oxidation of BAL under the influence of ACVA.

**Figure 12:**
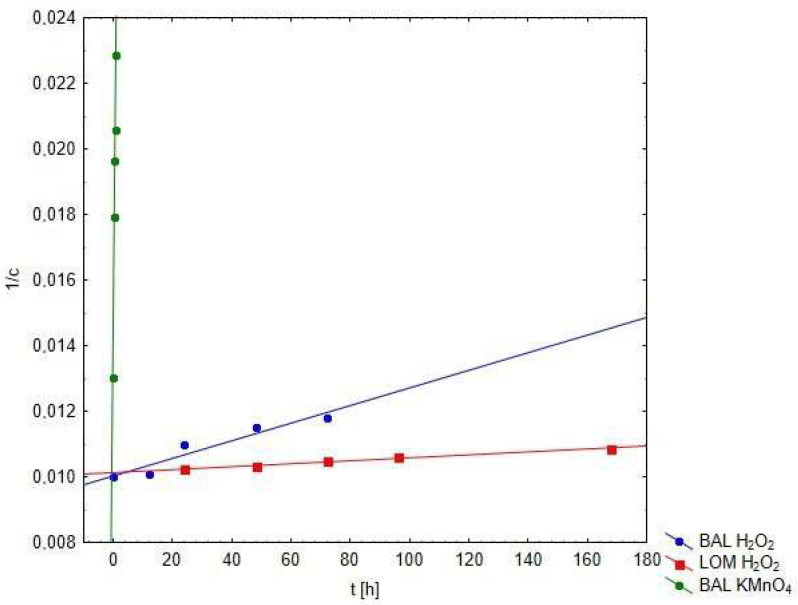
The 1/c = f(t) graph of oxidation of examined fluoroquinolones (FQs) by H_2_O_2_ and BAL by KMnO_4_.

The analysis of the plots 1/c = f(t) for both the oxidation of BAL and LOM while using ACVA and H_2_O_2_ demonstrated a highly significant correlation which confirmed that the oxidation process followed the kinetics of second order reaction (Figure 10, Figure 11 and Figure 12). The kinetic parameters of the oxidation reactions, such as degradation rate constant k and half-life t_0.5_ were calculated for all examined conditions (Table 2 and Table 3).

The calculated kinetic parameters for the oxidation process for individual FQs suggest that the rate of the oxidation process is dependent on the oxidation agent used, the type of FQ tested, temperature, and for a reaction involving ACVA also on the presence of methanol in the tested sample (Table 2 and Table 3).

Under the conditions of the experiment, the oxidation of LOM and BAL under the influence of ACVA proceeded faster with an increasing temperature. There were no differences in the rate of oxidation reaction for methanolic LOM and BAL solutions at the temperature of 60 °C, but they appeared at the temperatures of 50 °C and 40 °C. The kinetic parameters of the LOM oxidation process under the influence of ACVA also indicate that, for all tested temperatures, it runs faster for water solutions than for methanolic solutions (Table 2). The explanation for this phenomenon may be the study described in the work of Nelson at el. They suggested that, when using ACVA or AIBN as oxidants, alkoxy radicals are actually a significant source of the observed oxidizing reactivity. The presence of 5–10% methanol in the reaction mixture significantly quenches alkoxy radical reactivity, reducing the degradation rate of API. The observed effect is due to the preferential removal of H atoms from methanol and its trace impurities by alkoxy radicals [31].

The oxidation process of the tested FQs under the influence of hydrogen peroxide showed significant differences. The oxidation of BAL was about 5.8 times faster than that of LOM. When oxidizing LOM with hydrogen peroxide at RT, a significant difference was observed when compared to using ACVA at 40 °C. The oxidation of both the aqueous and methanolic solutions of LOM by ACVA proceeded much faster than the oxidation by H_2_O_2_. Additionally, while taking the oxidation of LOM by KMnO_4_ at pH 4.5 described in the literature into account [27], it can be concluded that this oxidizer causes that the process is much faster (t_0.5_ = 2.2 h) than under the influence of ACVA at 40, 50 and 60 °C.

In the case of BAL, oxidation under the influence of ACVA at 40 °C was slower than under the influence of H_2_O_2_ at RT; however, at the other temperatures of 50 °C and 60 °C, it was faster (Table 2 and Table 3). The oxidation process of BAL by KMnO_4_ in an acidic environment at RT was also investigated (Table 3). The obtained results indicate that oxidation by KMnO_4_, as in the case of LOM, is much faster (t_0.5_ = 1.1 h) than under the influence of H_2_O_2_ (t_0.5_ = 370.4 h) or ACVA at 60 °C (t_0.5_ = 100.0 h).

### 2.5. Identification of Oxidation Products

The identification of degradation products of examined FQs was performed on a basis of UHPLC-MS/MS analysis and supported by fragmentation patterns that were obtained from MS/MS experiments. The degradation products are shown in Table 4 and Table 5.

**Scheme 1:**
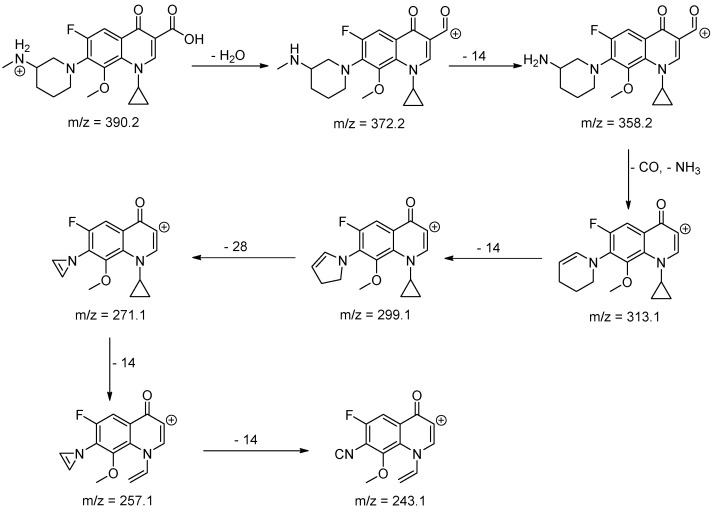
Proposed fragmentation pattern of BAL.

**Scheme 2:**
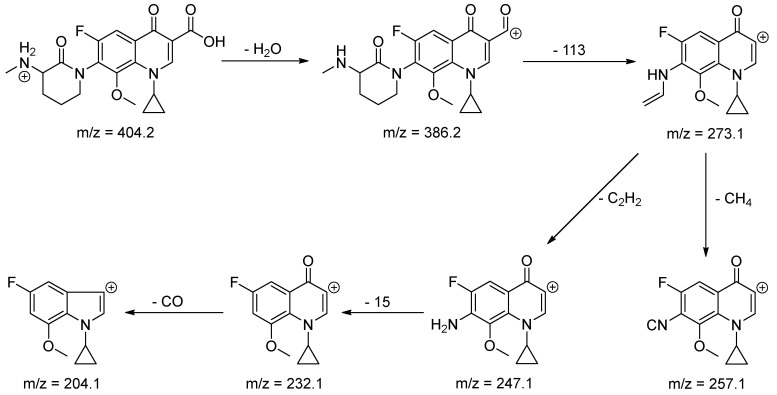
Proposed fragmentation pattern of **BP-1**.

**Scheme 3:**
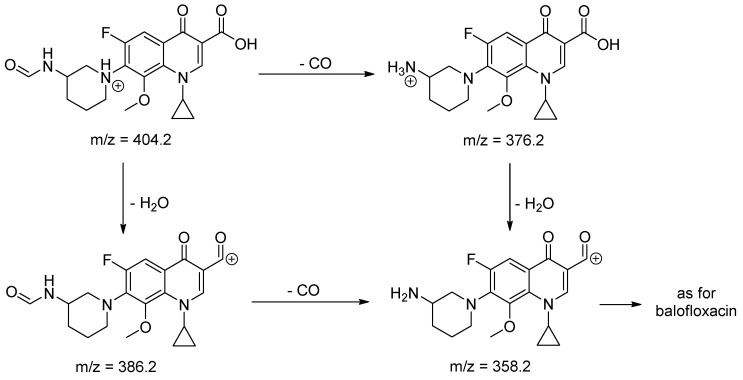
Proposed fragmentation pattern of **BP-2**.

**Scheme 4:**
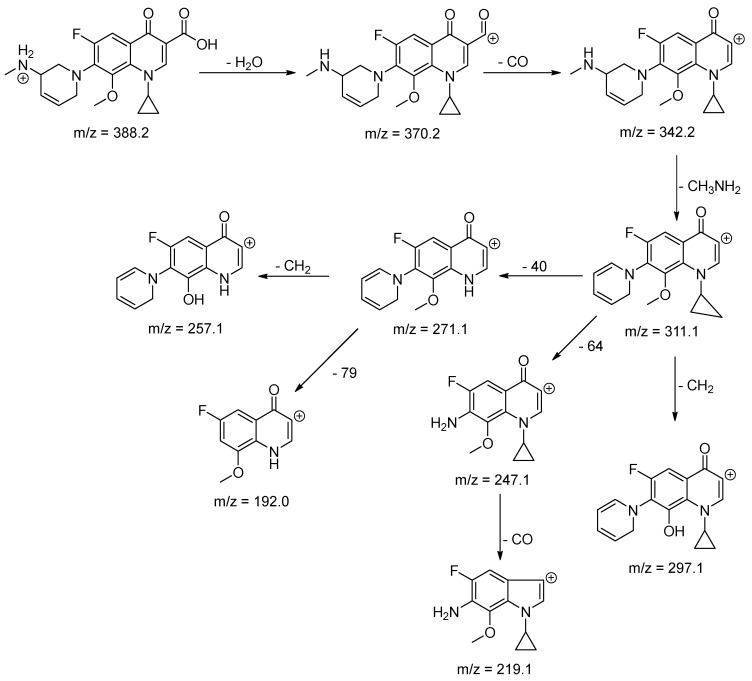
Proposed fragmentation pattern of **BP-3**.

**Scheme 5:**
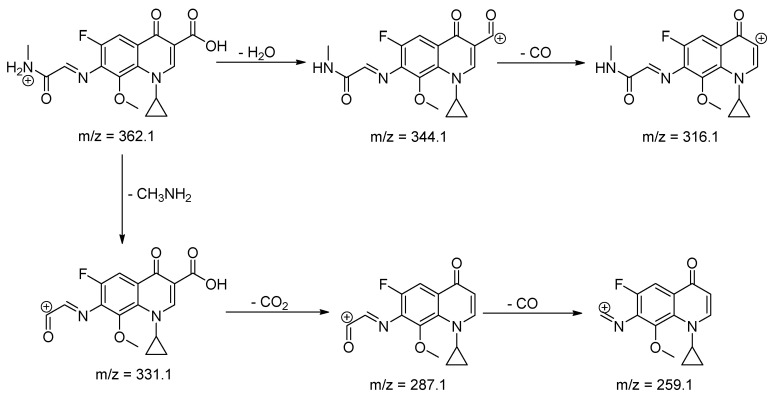
Proposed fragmentation pattern of **BP-4**.

**Scheme 6:**
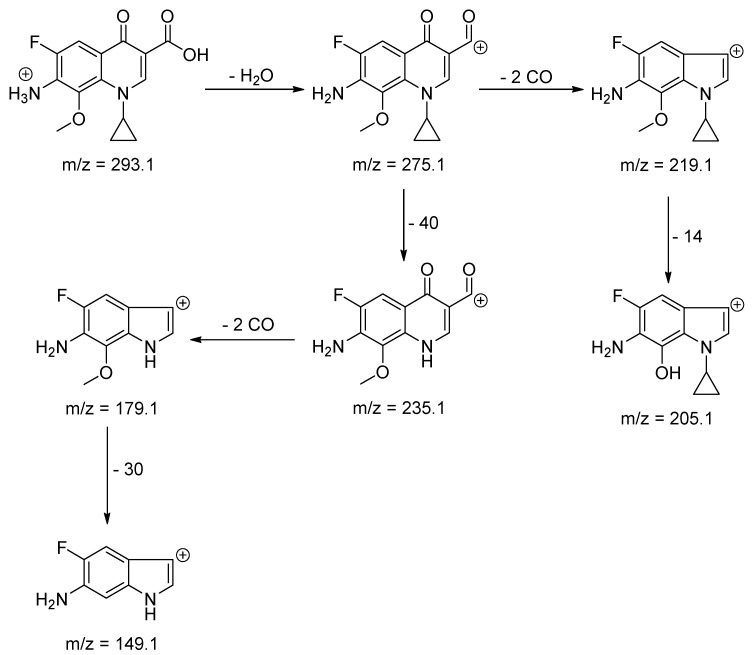
Proposed fragmentation pattern of **BP-5**.

In our previous work, we published the results of oxidation of LOM by KMnO_4_ [27]. In this study, the oxidation of BAL while using KMnO_4_ in an acidic medium at RT was also checked and it was possible to try to determine the most probable structures of oxidation products. These products were compared with those that were obtained by oxidation with ACVA and H_2_O_2_. The results are summarized in Table 4. The degradation process was found to affect 3-(methylamino)piperidine moiety of BAL. In the case of degradation with H_2_O_2_, it seems, that the oxidation of the ring led to its opening yielding **BP-4**. Degradation with KMnO_4_ yielded more products of 3-(methylamino)piperidine oxidation (**BP-1**–**BP-3**), leading finally to **BP-5**. Degradation with free radicals generating agent ACVA yielded similar products to KMnO_4_ oxidation, but in a lesser amount (**BP-3**, **BP-5**). The fragmentation patterns of BAL and its degradation products are shown in Scheme 1, Scheme 2, Scheme 3, Scheme 4, Scheme 5 and Scheme 6.

**Scheme 7:**
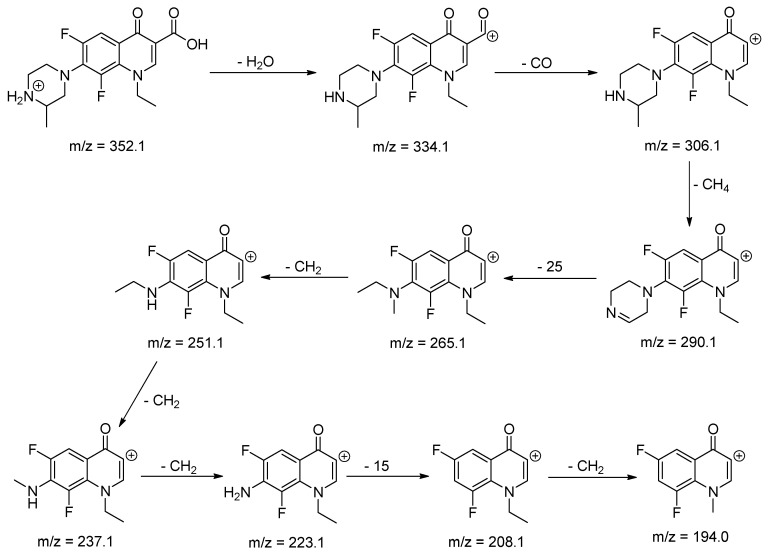
Proposed fragmentation pattern of LOM.

**Scheme 8:**
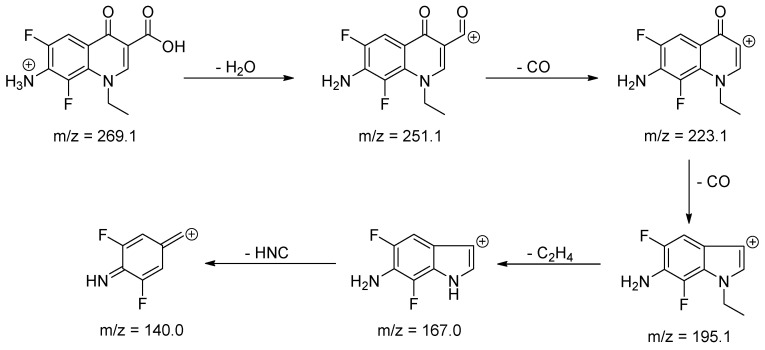
Proposed fragmentation pattern of **LP-1**.

**Scheme 9:**
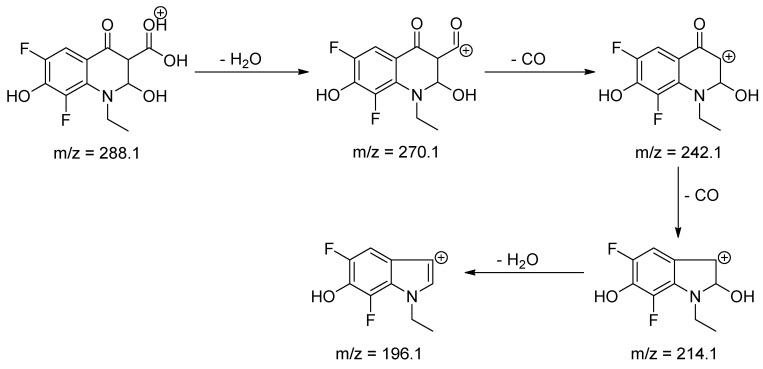
Proposed fragmentation pattern of **LP-2**.

**Scheme 10:**
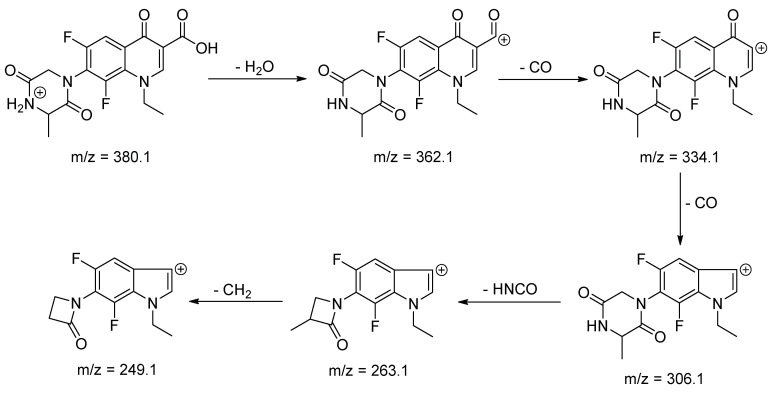
Proposed fragmentation pattern of **LP-3**.

In the case of LOM degradation process with ACVA was found to affect mainly 3-(methyl)piperazine moiety, leading to its oxidation (**LP-3**) with further ring cleavage (**LP-1** and **LP-2**). Additionally, for the product **LP-2** hydration of pyridin-4-one moiety was observed (Table 5). In the case of LOM degradation under the influence of H_2_O_2_, the chemical structure of the resulting product has not been established. The fragmentation patterns of LOM and its degradation products are shown in Scheme 7, Scheme 8, Scheme 9 and Scheme 10.

It may be assumed that the degradation with ACVA due to generation of ROS (Reactive Oxygen Species) yields similar products to those that were observed for KMnO_4_, but in a lesser amount. At the same time the degradation with H_2_O_2_ yields products with a lower degree of oxidation, probably due to the lower redox potential.

Additionally, the toxicity risk assessment of described degradation products was estimated based on their structure while using Osiris Property Explorer (https://www.organic-chemistry.org/). Fortunately, none of them show more risks than primal substance.

## 3. Materials and Methods

### 3.1. Chemicals and Reagents

Balofloxacin (Cat. No. SZBD078XV); Lomefloxacin hydrochloride (Cat. No. 129K2157) and (4,4′-azbis(4-cyanovaleric acid) (ACVA) were purchased from Sigma–Aldrich (Steinheim, Germany). HPLC grade methanol and acetonitrile were purchased from WITKO (Łódź, Poland). Hydrogen peroxide 30%, sodium dihydrogen phosphate anhydrous and orthofosforic acid (85%), and HPLC grade water were purchased from Merck (Darmstadt, Germany).

### 3.2. Standard Solution

The amount of 0.1000 g of each FQs was weighed on analytical balance. LOM was dissolved in the volume of 50 mL of water or methanol, and filled up to the 100 mL with the same solvent. The weighed sample of BAL was dissolved in the volume of 80 mL of methanol, and then filled up to the 100 mL with the same solvent.

For method validation, solutions containing different concentrations of the examined FQs in the range 0.05–0.18 mg·mL^−1^ for LOM and 0.05–0.26 mg·mL^−1^ for BAL were prepared.

### 3.3. HPLC—DAD Analysis

The liquid chromatography system, HITACHI, High-Technologies Corporation (Tokyo, Japan) equipped with a solvent delivery pump (L-2130), degasser, an autosampler (L-2200), a photodiode array detector (L-2455), and a column oven (L-2350) was used. The chromatographic analysis of chosen substances was performed on Kinetex 5u XB—C18 100A column, Phenomenex (Torrance, CA, USA) (250 × 4.6 mm, 5 μm particle size, core shell type), coupled with guard-column. The column temperature was 30 °C. The chromatographic separation was achieved using as a mobile phase mixture of 0.05 M phosphate buffer (pH = 3.20 adjusted with *o*-phosphoric acid) and acetonitrile (87:13 *v/v* for LOM; 80:20 *v/v* for BAL). The flow rate of the mobile phase was 1.0 mL∙min^−1^, and the injection volume was 10 µL. The analysis time was 55 min for LOM and 40 min for BAL. The samples were monitored at 280 nm for LOM and at 295 nm for BAL.

### 3.4. UPLC/MS/MS Analysis

The UPLC-MS/MS system consisted of a Waters ACQUITY UPLC (Waters Corporation, Milford, MA, USA) coupled to a Waters TQD mass spectrometer (electrospray ionization mode ESI-tandem quadrupole). Chromatographic separations were carried out using the Acquity UPLC BEH (bridged ethyl hybrid) C_18_ column; 2.1 × 100 mm, and 1.7 µm particle size, equipped with Acquity UPLC BEH C18 VanGuard pre-column; 2.1 × 5 mm, and 1.7 µm particle size. The column was maintained at 40 °C and eluted under gradient conditions using from 95% to 0% of eluent A over 10 min, at a flow rate of 0.3 mL∙min^−1^. Eluent A: 0.1% water solution of formic acid; eluent B: 0.1% solution of formic acid in acetonitrile. Chromatograms were recorded while using Waters eλ PDA detector. The spectra were analyzed in 200–700 nm range with 1.2 nm resolution and sampling rate 20 points/s.

MS detection settings of Waters TQD mass spectrometer were as follows: source temperature 150 °C, desolvation temperature 350 °C, desolvation gas flow rate 600 L·h^−1^, cone gas flow 100 L·h^−1^, capillary potential 3.00 kV, and cone potential 30 V. Nitrogen was used for both nebulizing and drying gas. The data were obtained in a scan mode ranging from 50 to 1000 *m/z* in time 0.5 s intervals; eight scans were summed up to get the final spectrum. In MS/MS experiments collision energy was set to 50 eV and argon was used as a collision gas. Data acquisition software was MassLynx V 4.1 (Waters (Milford, MA, USA)).

### 3.5. Method Validation

The proposed method was validated for the determination of BAL and LOM in the presence of oxidation products by HPLC analysis according to ICH guidelines [6]. In order to assess the specificity of the new HPLC methods the 0.5 mM solutions of BAL and LOM after reaction with chosen oxidizers were analyzed. Oxidation study was performed in 10 mM ACVA solution heated at 50 °C; the solution was left for 48 h, in 3% H_2_O_2_ after 72 h of incubation and in acidic medium with the presence of KMnO_4_ after 1 h of incubation.

The linearity for BAL and LOM was determined preparing five solutions that covered the concentration range of 0.05–0.26 mg·mL^−1^ for BAL and 0.05–0.18·mg·mL^−1^ for LOM. The correlation coefficient, determination coefficient, slope of the regression line and standard deviation of slope, y-intercept and standard deviation of intercept, and also standard error of residuals of the calibration curve were calculated while using Statistica 13.3 (TIBCO Software Inc., Palo Alto, CA, USA). The Shapiro–Wilk statistical test was also done. Based on the formula LOD = 3.3 S_e_/a and LOQ = 10 S_e_/a, the LOD and LOQ for examined FQs was calculated. The values of LOD and LOQ were then experimentally confirmed.

The six separately prepared solutions were analyzed to check the precision of the method at the concentration level 0.10 mg·mL^−1^ for BAL and LOM. The same procedure was followed for three another days by different analysts to examine the intermediate precision of the described methods. The precision and the intermediate precision were then assumed by the calculated RSD (%) value of the peak area of tested FQs. The contents of mobile phase were made intentionally in order prove the robustness of the proposed methods small modifications of flow rate. To check the effect of the flow rate on resolution, the flow rate was changed to 1.10 and 0.90 mL·min^−1^ (primary flow rate was 1.0 mL·min^−1^). The temperature of the column was modified to 28 °C and 32 °C (instead of 30 °C), and the composition of the mobile phase was changed ±5% from the initial value.

### 3.6. Preparation of Samples and Execution of Oxidation Tests with the Participation of ACVA

The test samples were prepared, as follows: the appropriate volume of a 1 mg·mL^−1^ solutions of BAL or LOM was added to 25 mL volumetric flask and filled up with 10 mM water-acetonitrile (50:50 *v/v*) solution of ACVA to obtain a concentration of FQ of 0.5 mM. Next, 2.0 mL of this prepared solution was added to the 2.0 mL vials. Additionally, an ACVA control samples that did not contain FQ and control samples of fluoroquinolone without ACVA solution were prepared in 2.0 mL vials. All of the samples were then closed tightly with a stopper and a cap and incubated in heating blocks (QBH_2_ Grant, Grant Instruments), set at temperatures 40 °C, 50 °C and 60 °C. The samples of BAL were incubated at 40 °C for 144 h; at 50 °C and 60 °C for 48 h. The samples of LOM (water solution) were incubated at 40 °C for 96 h, at 50 °C for 72 h and at 60 °C for 36 h. The samples of LOM (methanolic solution) were incubated at 40 °C for 216 h, at 50 °C for 96 h and at 60 °C for 48 h. At appropriate time intervals which were mainly dependent on the type of FQ and temperature, the content of one vial was analyzed in duplicate.

### 3.7. Preparation of Samples and Execution of Oxidation Tests with the Participation of 3% H_2_O_2_

The test samples were prepared, as follows: the appropriate volume of a 1.0 mg·mL^−1^ solution of BAL or LOM was added to 25 mL volumetric flask and filled up to the final volume with 3% H_2_O_2_ solution to obtain the final concentration of FQ of 0.5 mM. Next, 2.0 mL of this prepared solution was added to the 2.0 mL vials.

All of the samples were then closed tightly with a stopper and a cap and incubated in room temperature. The samples of BAL were incubated at RT for 72 h. The samples of LOM (water solution) were incubated at RT for 168 h. At appropriate time intervals which were mainly dependent on the type of FQ and temperature, the content of one vial was analyzed. The analyses were performed in duplicate.

### 3.8. Preparation of Sample of BAL and Execution of Oxidation Tests with the Participation of KMnO_4_

The sample of BAL was prepared in the same way described in our work regarding the influence of KMnO_4_ on the LOM [27]. An acetate buffer pH 4.5 was used in the study. After 15, 30, 45, and 60 min of incubation at RT, the tested samples were analyzed by HPLC.

### 3.9. Determination of Kinetic Parameters of the Oxidation Process

In order to determine the order of the reaction, ln c or 1/c dependence curves were plotted against the incubation time, and then the fit to the linear model was checked and the correlation coefficients were determined. The symbol c represents the percent of remaining non-degraded FQ. Subsequently, the reaction rate constants (k) and degradation times of 50% for the tested FQs (t_0.5_) were calculated.

## 4. Conclusions

The oxidation process of BAL and LOM under the influence of an azonitrile radical initiator, i.e., ACVA at 40, 50, and 60 °C, and hydrogen peroxide were investigated. Additionally, the oxidation of BAL under the influence of KMnO_4_ in an acidic environment was assessed. The oxidation process was estimated while using newly developed HPLC methods that were validated. The oxidation reactions in the presence of ACVA, H_2_O_2_, and KMnO_4_ followed the kinetics of the second-order reactions. Based on the conducted research and literature data [27], it was found that the FQs oxidation process is the fastest as a result of KMnO_4_ in an acidic environment. The profile of the resulting degradation products of BAL is similar to the profile that was obtained under ACVA. The profiles of LOM degradation products produced under the influence of KMNO_4_ [27] and ACVA were different. Whereas under the influence of hydrogen peroxide, the most frequently used in drug stability studies, only one product was formed. While using the UHPLC-MS/MS method, the probable structures of the oxidation products of the tested FQs were established, which were not previously described in the literature.

It seems that the use of other oxidants in stress tests, apart from H_2_O_2_, is fully justified and should be recommended due to the obtained results. In addition to the value of scientific knowledge, the research undertaken is of practical importance for the technology of the drug form, the aim of which is to obtain stable, safe, and effective drugs.

## Figures and Tables

**Table 1:** Validation of the method.

Parameter	BAL	LOM
t_R_ (min) ^a^	10.03 ± 0.41	14.50 ± 0.27
LOD (mg·mL^−1^)	0.0117	0.0138
LOQ (mg·mL^−1^)	0.0355	0.0419
Linear range (mg·mL^−1^)	0.05–0.26	0.05–0.18
Regression equation (y): Slope (a ± S_a_)	1861 × 10^5^ ± 2,842,634	1708 × 10^5^ ± 4,792,179
Intercept (b ± S_b_)	−989,217 ± 490,253	1,078,726 ± 581,021
t = b/S_b_	−2.02 < t_α,f_	1.86 < t_α,f_
Normality of residuals ^b^ (Shapiro-Wilk test)	0.9319 (*p* = 0.47)	0.9399 (*p* = 0.49)
Correlation coefficient r	0.9991	0.9961
Determination coefficient r^2^	0.9981	0.9922
Precision (% RSD)	0.17	0.38
Intermediate precision (% RSD)	0.64	0.83

^a^ Mean ± SD (*n* = 6); LOD—limit of detection, LOQ—limit of quantification; Regression equation y = ac + b; c—concentration of solution; y—peak area; S_a_—standard deviation of slope; S_b_—standard deviation of intercept; t—calculated value of Student’s *t*-test, t_α,f_ = 2.776 critical value of Student’s t-test for degrees of freedom f = 4 and significance level α = 0.05; ^b^ normal distribution of residuals if *p* > 0.05.

**Table 2:** The kinetic results of the FQs oxidation by ACVA.

Component	Temperature	k (h^−1^)	t_0.5_ (h)	r
BAL (methanol solution)	40 °C	0.13 × 10^−4^	769.2	0.9920
	50 °C	0.42 × 10^−4^	238.1	0.9985
	60 °C	0.10 × 10^−3^	100.0	0.9944
LOM (water solution)	40 °C	0.14 × 10^−4^	714.3	0.9874
	50 °C	0.10 × 10^−3^	100.0	0.9940
	60 °C	0.13 × 10^−3^	76.9	0.9843
LOM (methanol solution)	40 °C	0.11 × 10^−4^	909.1	0.9893
	50 °C	0.58 × 10^−4^	172.4	0.9568
	60 °C	0.10 × 10^−3^	100.0	0.9778

k—rate constant, r—correlation coefficient.

**Table 3:** The kinetic results of the oxidation of FQs by H_2_O_2_ and KMnO_4_.

Component	Temperature	Oxidizer	k (h^−1^)	t_0.5_ (h)	r
BAL	RT	KMnO_4_	8.92 × 10^−3^	1.1	0.9566
		H_2_O_2_	0.27 × 10^−4^	370.4	0.9554
LOM	RT	H_2_O_2_	0.46 × 10^−5^	2173.9	0.9928

k—rate constant, r—correlation coefficient, RT—room temperature.

**Table 4:** Proposed structures of the degradation products of BAL.

Compound Oxidizer	t_R_^a^ (min)	[M + H^+^]	Fragmentation Ions	Structure
**BP-1** KMnO_4_	2.34	404.2	204.1, 232.1, 247.1, 257.1, 273.1, 386.2	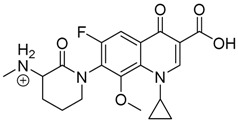
**BP-2** KMnO_4_	2.91	404.2	243.1, 257.1, 271.1, 299.1, 313.1, 358.2, 372.2, 386.2	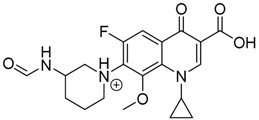
**BP-3** KMnO_4_ ACVA	3.03	388.2	192.0, 219.1, 247.1, 257.1, 271.1, 297.1, 311.1, 342.2	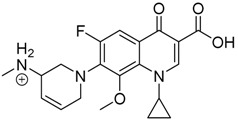
**BP-4** H_2_O_2_	3.10	362.1	259.1, 287.1, 316.1, 331.1, 344.1	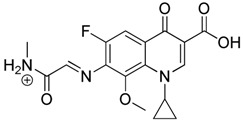
**BAL**	3.48	390.2	243.1, 257.1, 271.1, 299.1, 313.1, 358.2, 372.2	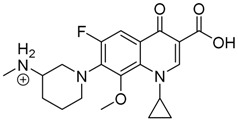
**BP-5** ACVA KMnO_4_	4.25	293.1	149.1, 179.1, 205.1, 219.1, 235.1, 275.1	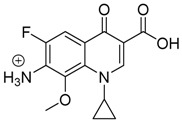

**^a^** retention times recorded by UHPLC-MS/MS.

**Table 5:** Proposed structures of the degradation products of LOM formed under the influence of ACVA.

Compound	t_R_ (min)	[M + H^+^]	Fragmentation Ions	Structure
**LOM**	2.92	352.1	194.0, 208.1, 223.1, 237.1, 251.1, 265.1, 290.1, 306.1, 334.1	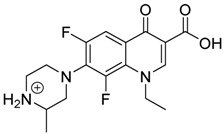
**LP-1**	4.10	269.1	140.0, 167.0, 195.1, 223.1, 251.1	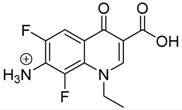
**LP-2**	4.71	288.1	196.1, 214.1, 242.1, 270.1	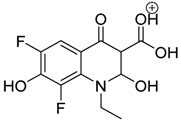
**LP-3**	4.76	380.1	249.1, 263.1, 306.1, 334.1, 362.1